# TiFe_2_O_4_@SiO_2_–SO_3_H: A novel and effective catalyst for esterification reaction

**DOI:** 10.1016/j.heliyon.2024.e26286

**Published:** 2024-02-10

**Authors:** Mohanad Yakdhan Saleh, Ahmed Kareem Obaid Aldulaimi, Shakir Mahmood Saeed, Ayat Hussein Adhab

**Affiliations:** a Department of Chemistry, College of Education for Pure Science, University of Mosul, Mosul, Iraq; b College of Food Sciences, Al-Qasim Green University, Babylon, Iraq; c Department of Pharmacy, Al-Noor University College, Nineveh, Iraq; d Department of Pharmacy, Al-Zahrawi University College, Karbala, Iraq

**Keywords:** TiFe_2_O_4_, Palmitic acid, SO_3_H, Catalyst

## Abstract

In the present study, TiFe_2_O_4_@SiO_2_–SO_3_H heterogeneous catalyst was successfully synthesized and applied to generate biodiesel from oleic acid, and palmitic acid using an esterification process. In this sense, the nanocatalyst surface was characterized using TEM, TGA, XRD, FTIR, VSM, BET, SEM, and EDX analyses. Nanocatalyst TiFe_2_O_4_@SiO_2_–SO_3_H showed high activity for the esterification of oleic acid and palmitic acid. Also, the nanocatalyst can be easily recovered with a bar magnet and reused many times without any loss of activity.

## Highlights

• This is the first report of this catalyst.
• Nanocatalyst can be recovered and reused without decreasing catalytic activity.
• Nanocatalyst characterized by FT-IR, EDS, SEM, TGA, XRD, TEM, BET, and VSM technique.
• Non-toxic content of the described catalyst.

## 1 Introduction

Esterification of alcohols with carboxylic acids has recently attracted the attention of scientists for the industrial production of useful chemicals such as perfumes, biodiesel, polymers, solvents, etc. [[1], [2], [3], [4]]. In the next decade, due to the reduction of non-renewable fossil fuels and energy consumption, biodiesel will be a suitable alternative in the industry [[5], [6], [7], [8]] [[5], [6], [7], [8]] [[5], [6], [7], [8]]. Biodiesel is a renewable, clean, sulfur-free, and sustainable fuel derived from monoalkyl esters of fatty acids. Biodiesel is usually produced by the esterification of fatty acids in non-edible or edible oils with primary alcohols using basic or acidic catalysts [9,10]. Also, esterification of free fatty acids such as palmitic acid and oleic acid in incompatible raw materials before using basic catalysts is important for the transesterification reaction due to soap formation, therefore acid nanocatalysis is more suitable for biodiesel production [11]. Therefore, using an acid catalyst, environmentally friendly and green biodiesel can be produced from palmitic acid and oleic acid [12,13].

The development and research of green and efficient nanomaterials as catalyst supports is a big challenge in the synthesis of organic compounds [[14], [15], [16]] [[14], [15], [16]] [[14], [15], [16]]. In the last decade, magnetic nanoparticles have been recognized as excellent supports due to their easy preparation and operation, and high surface area, easy recovery by the magnetic field, which will lead to increased product purity [17,18]. Also, the most important advantage of magnetic nanoparticles is their separation from the reaction mixture with the help of a bar magnet [19]. Among the heterogeneous nanocatalysts, TiFe_2_O_4_ has received much attention due to its simplicity in the synthesis method and easy separation using magnets [20,21]. However, various catalysts can be supported on TiFe_2_O_4_ nanoparticles, because they can be easily separated after several consecutive uses in the reaction [22,23].

In this work, we report the synthesis and structural characterization of a green and novel catalyst and investigate their utility as a green and efficient catalyst in Biodiesel Production. Compared to previously reported catalysts, this solid acid catalyst showed better catalytic performance.

## 2 Experimental

### 2.1 Preparation of TiFe_2_O_4_@SiO_2_–SO_3_H

**Scheme 1:**
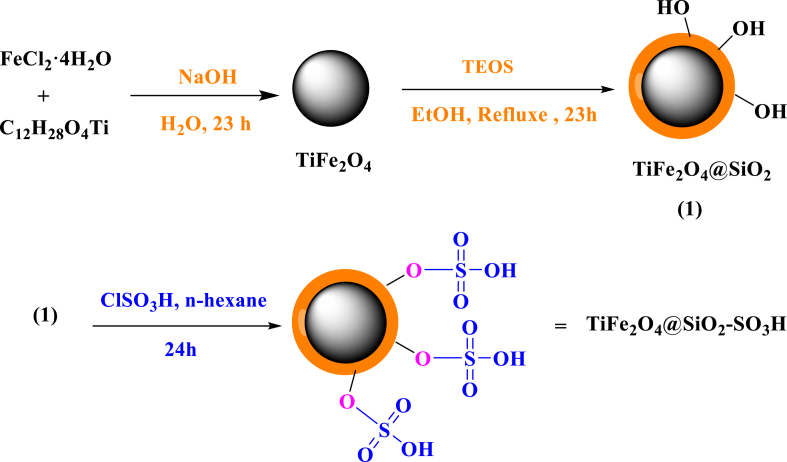
Synthesis of TiFe_2_O_4_@SiO_2_–SO_3_H

To synthesize the TiFe_2_O_4_ NPs, 10 mmol of Titanium isopropoxide (C_12_H_28_O_4_Ti) and 20 mmol of FeCl_3_.4H_2_O were prepared and the mixture was maintained at 70 °C water bath for 30 min. Next, 5 g of sodium hydroxide was added and the mixture was stirred. The resulting particles were harvested, washed several times using H_2_O, and dried at 100 °C (Scheme 1). Next, 2.0 g of the obtained TiFe_2_O_4_ was dispersed in a mixture of ethanol (70 mL), 10.0 mL of ammonia solution, 20 mL of H_2_O, followed by the addition of 5 g of PEG, and 2.5 mL of TEOS (tetraethyl orthosilicate). This solution was stirred for 24 h at room temperature. Also, the product (TiFe_2_O_4_@SiO_2_) was separated using a simple magnet washed several times with ethanol and water, and dried at room temperature. Finally, to prepare the TiFe_2_O_4_@SiO_2_–SO_3_H catalyst, a mixture of TiFe_2_O_4_@SiO_2_ (2.0 g) was dispersed in 100 ml of hexane in a round-bottomed flask. In the next step, 0.3 g of chlorosulfonic acid was added drop by drop to the reaction vessel and finally stirred at 25 ^°^C for 24 h. Next, after completion of the reaction, the final catalyst (TiFe_2_O_4_@SiO_2_–SO_3_H) was separated and washed with H_2_O and ethanol and, dried under vacuum at 55 °C (Scheme 1).

### 2.2 Biodiesel production

**Scheme 2:**
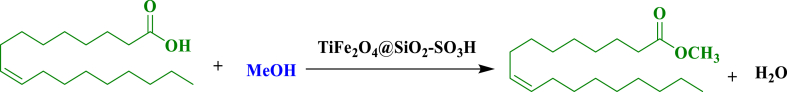
Esterification of oleic acid.

The catalytic activity of TiFe_2_O_4_@SiO_2_–SO_3_H was used for the esterification reactions of oleic acid. Therefore, methanol (9 mmol), oil (2 mmol), and nanocatalyst (0.02 g) were mixed in a round-bottom flask. Afterward, the mixture was heated at 60 °C for 1.5 h. The nanocatalyst was separated using a magnet after the completion of the reaction, and excess methanol was removed from the upper liquid phase using rotary evaporation. The extracted organic phase was washed with distilled water to remove the remaining impurities and finally, sodium sulfate was used to dry the organic phase (Scheme 2).

**Scheme 3:**
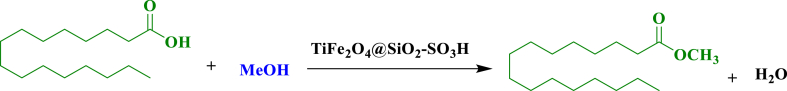
Esterification of Palmitic acid.

The catalytic activity of TiFe_2_O_4_@SiO_2_–SO_3_H was used for esterification reactions of Palmitic acid. Therefore, Palmitic acid (2 mmol), methanol (9 mmol), and TiFe_2_O_4_@SiO_2_–SO_3_H (catalyst) (0.02 g) were mixed in a round-bottom flask (Scheme 3). Afterward, the mixture was heated at 70 °C for 1 h. After the completion of the reaction, TiFe_2_O_4_@SiO_2_–SO_3_H was separated using an external magnet, and excess methanol was removed from the upper liquid phase using rotary evaporation. Then, to remove impurities, the organic phase was washed with distilled water and dried using Na_2_SO_4_.

### 2.3 Selected NMR data

**S**_**1**_**) Methyl oleate**:^1^H NMR (CDCl_3_, 400 MHz): δ = 0.83 (s, 3H, CH_3_), 1.11 (m, 20H, 10CH_2_), 1.36 (m, 2H, CH_2_), 1.89 (m, 4H, 2CH_2_), 2.35 (t, 2H, CH_2_), 3.56 (s, 3H, CH_3_), 5.59 (m, 2H, 2CH) ppm. FT-IR (KBr) cm^−1^: 589, 720, 1202, 1463, 1752, 2953.

**S**_**2**_**) methyl palmitate**:^1^H NMR (CDCl_3_, 400 MHz): δ = 0.83 (s, 3H, CH_3_), 1.03 (m, 24H, 12CH_2_), 1.49 (m, 2H, CH_2_), 2.19 (m, 2H, CH_2_), 4.15 (s, 3H, CH_3_) ppm. FT-IR (KBr) cm^−1^: 604, 723, 1208, 1743, 2884, 3426.

#### 2.3.1 Catalyst characterizations

**Fig. 1:**
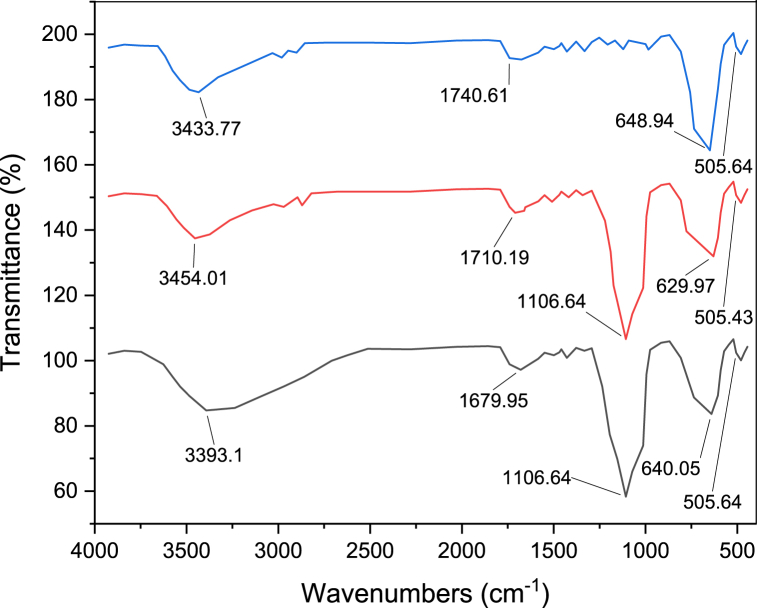
Comparative study of FTIR spectra of a) TiFe_2_O_4_, b) TiFe_2_O_4_@SiO_2_, c) TiFe_2_O_4_@SiO_2_–SO_3_H

FT-IR spectra of TiFe_2_O_4_ (a), TiFe_2_O_4_@SiO_2_ (b), and TiFe_2_O_4_@SiO_2_–SO_3_H (c) catalyst are shown in Fig. 1. The FT-IR spectrum of TiFe_2_O_4_ nanoparticles shows two bands in the regions of 505 and 648 cm^−1^ are assigned to the stretching vibrations of the titanium–oxygen and the iron-oxygen bonds, respectively. In Fig. 1b, the observation of the stretching vibration band at 1106 cm^−1^ is related to Si–O bonds and evidence for the presence of SiO_2_ on the surface of TiFe_2_O_4_ nanoparticles. In Fig. 1c, the functionalization of –SO_3_H groups on TiFe_2_O_4_@SiO_2_ was approved by the absorption of OH stretching bands of the –SO_3_H moiety at 2500–3500 cm-1 in the FT-IR spectrum [24].

**Fig. 2:**
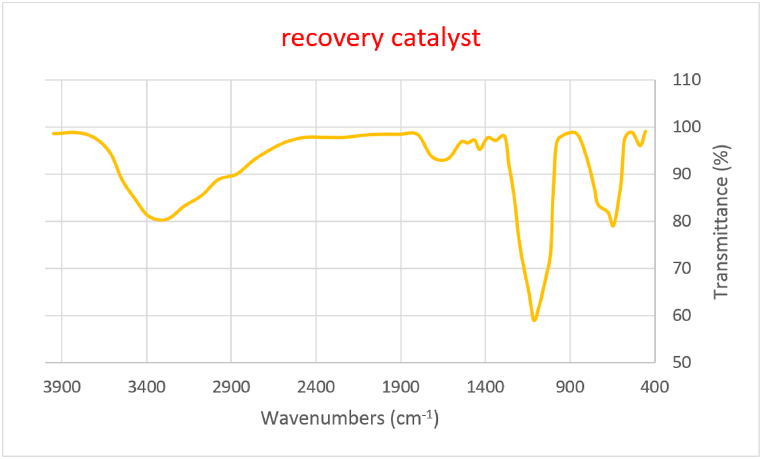
FTIR spectra of recovery TiFe_2_O_4_@SiO_2_–SO_3_H

Fig. 2 shows the FT-IR spectrum of the nanocatalyst after recycling. There is no change in the FT-IR of TiFe_2_O_4_@SiO_2_–SO_3_H after recovery, which confirms the stability of the nanocatalyst (Fig. 2).

**Fig. 3:**
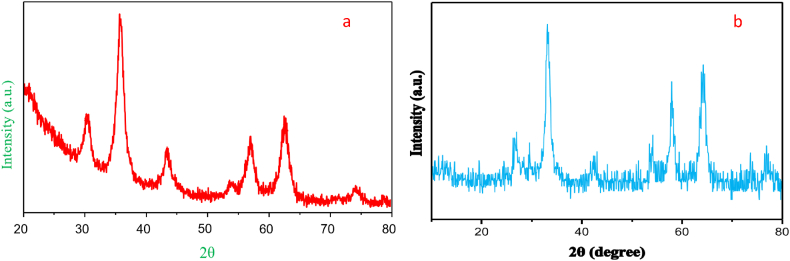
XRD spectrum of a) TiFe_2_O_4_ and b) TiFe_2_O_4_@SiO_2_–SO_3_H

The XRD pattern of TiFe_2_O_4_@SiO_2_–SO_3_H nanocomposite was presented in Fig. 3a and b. As shown in Fig. 3a, the TiFe_2_O_4_@SiO_2_–SO_3_H MNPs afforded seven sharp and strong peaks at 2θ = 30.1, 35.45, 43.12, 53.27, 56.88 and 62.45 indexed to the (2 2 0), (3 1 1), (4 0 0), (4 2 2), (5 1 1) and (4 4 0) planes, respectively showing good agreement with XRD pattern of previous reports on TiFe_2_O_4_ MNPs. These analyzes confirm that the TiFe_2_O_4_ structure is not degraded by the silica sulfuric acid shell stabilization, and that the background noise is caused by the dried amorphous SO_3_H shells (Fig. 3b) [25].

**Fig. 4:**
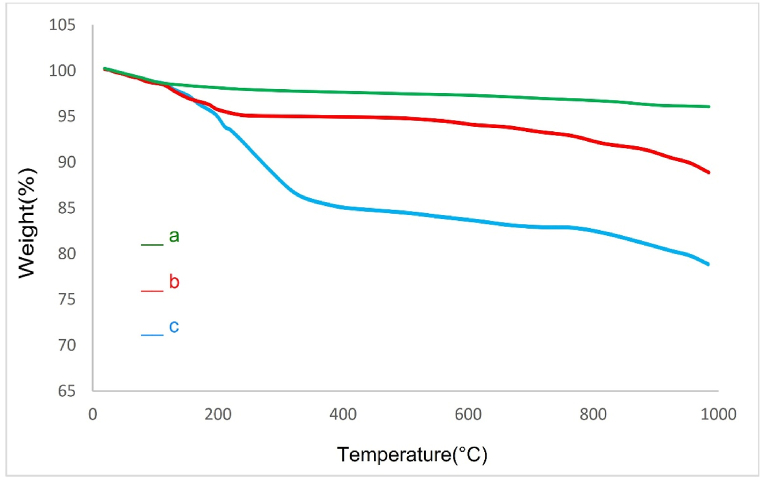
TGA curve of a) TiFe_2_O_4_, b) TiFe_2_O_4_@SiO_2_, c) TiFe_2_O_4_@SiO_2_–SO_3_H.

The TGA was investigated for the quantitative determination of the ligand (SiO_2_–SO_3_H) supported on the surface of TiFe_2_O_4_ magnetic nanoparticles (Fig. 4). As illustrated in Fig. 4a, the curve of TiFe_2_O_4_, the first change which is observed below 200 ^°^C may have corresponded to the loss of physically adsorbed H_2_O on the surface of this compound. The little quantity of weight loss (6%) after 200 ^°^C is due to the removal of SiO_2_ groups (Fig. 4b). As shown in Fig. 4c, for TiFe_2_O_4_@SiO_2_–SO_3_H, there is a weight loss of 11% between 250 and 700 ^°^C related to the breakdown of the TiFe_2_O_4_@SiO_2_–SO_3_H moieties. The results of the TGA analysis confirmed the successful support of SiO_2_–SO_3_H on the surface of TiFe_2_O_4_ MNPs.

**Fig. 5:**
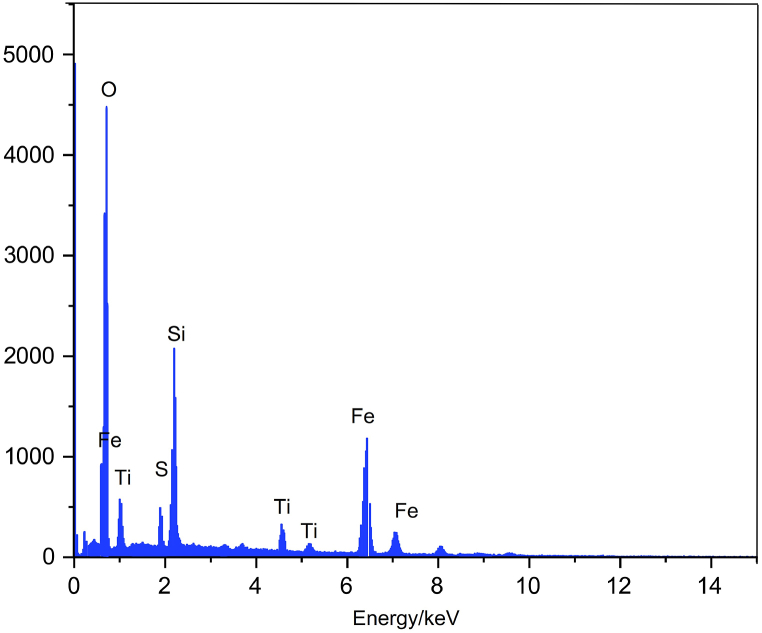
EDX images of TiFe_2_O_4_@SiO_2_–SO_3_H.

The EDX image of TiFe_2_O_4_@SiO_2_–SO_3_H nanocatalyst (Fig. 5) shows the presence of Ti, Si, Fe, S, and O elements. These results show that there are no other impurities related to the solvents and materials used in the catalyst synthesis steps.

**Fig. 6:**
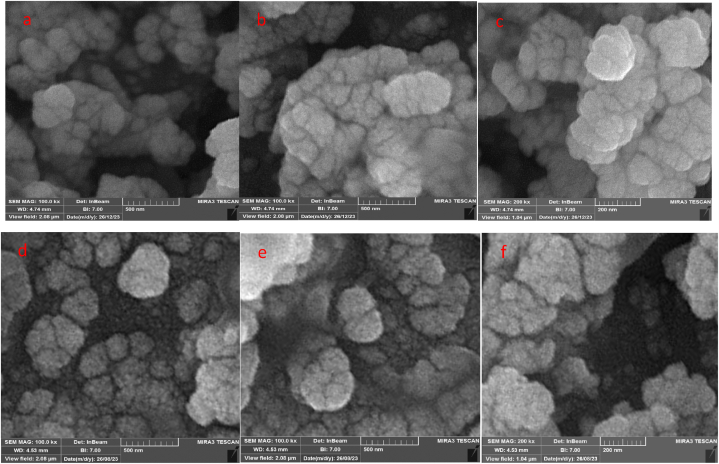
SEM images of TiFe_2_O_4_ (a–c), TiFe_2_O_4_@SiO_2_–SO_3_H (d–f).

The morphology of the nanoparticles in the scanning electron microscope (SEM) images (Fig. 6) shows that the nanoparticles are spherical and have a relatively uniform distribution. The SEM images of TiFe_2_O_4_ (Fig. 6a, b, and 6c) and TiFe_2_O_4_@SiO_2_–SO_3_H (Fig. 6d, e, and 6f) declared that the catalyst was synthesized as nanometer-sized quasi-spherical particles with 60–100 nm average diameter (Fig. 6). Also, a continuous layer of the SiO_2_–SO_3_H can be observed on the surface of the catalyst, if we compare TiFe_2_O_4_@SiO_2_–SO_3_H result with TiFe_2_O_4_.

**Fig. 7:**
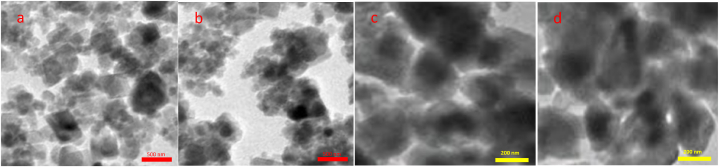
TEM images of TiFe_2_O_4_ (a and b), and TiFe_2_O_4_@SiO_2_–SO_3_H (c and d).

TiFe_2_O_4_ (Fig. 7a and b) and TiFe_2_O_4_@SiO_2_–SO_3_H (Fig. 7c and d) were characterized by TEM. The scanning electron microscopy images show that the size of the nanocatalyst particles is in the nanometer range (60–100 nm) with a sphere-like structure. Transmission electron microscopy images confirmed these observations (Fig. 7).

**Fig. 8:**
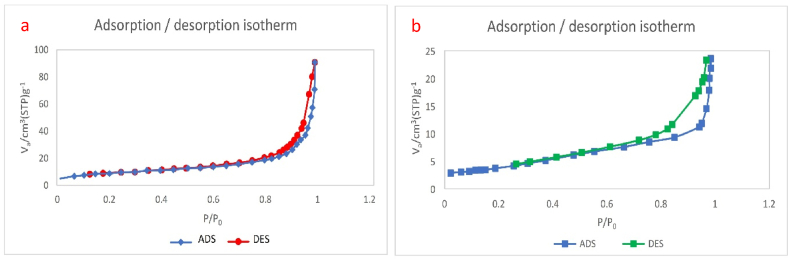
Nitrogen adsorption-desorption isotherm for TiFe_2_O_4_ (a) and TiFe_2_O_4_@SiO_2_–SO_3_H (b).

The adsorption–desorption isotherms of N_2_ at 77 K were characterized by porosity adsorption (Fig. 8). BET analysis was performed to know the mean pore diameter, total pore volume, and surface area of TiFe_2_O_4_ and TiFe_2_O_4_@SiO_2_–SO_3_H. In Fig. 8a, the N_2_ adsorption–desorption isotherm of TiFe_2_O_4_ has been displayed. Regarding the N_2_ adsorption-desorption isotherms technique, the obtained surface area of TiFe_2_O_4_ is 100.39 (m^2^/g). As shown in Fig. 8a, the size distribution and pore volumes of TiFe_2_O_4_ obtained were 0.34 cm^3^ g^−1^ and 5.7 nm respectively. Also, in Fig. 8b, the N_2_ adsorption–desorption isotherm of TiFe_2_O_4_@SiO_2_–SO_3_H can be observed. Regarding the N_2_ adsorption-desorption isotherms, based on the BET method, the obtained surface area of TiFe_2_O_4_@SiO_2_–SO_3_H is 21.01 (m^2^/g). Also, the total pore volumes and mean pore diameter of TiFe_2_O_4_@SiO_2_–SO_3_H were 0.15 cm^3^ g^−1^, and 16 nm, respectively. The reduction of the surface area in the final catalyst is due to the successful fixation of SO_3_H on the surface of TiFe_2_O_4_ (Fig. 8a and b).

In the next step, using the back-titration method, the acid strength of the synthesized catalyst, that is, the surface density of SO_3_H groups, was determined. First, 0.1 g of the synthesized catalyst was dispersed in 60 ml of water in a flask and stirred for 30 min, then 10 ml of NaOH (0.1 N) was added to the reaction vessel under constant stirring and until the pH changed. did not change. The catalyst was separated using an external magnet. Then, two drops of phenolphthalein were added to the container and were tittered with 1.1 ml HCl (0.1 N). Thus 1 g of catalyst has 8.9 mmol of the acidic group which is higher than the amount reported so far in the literature.

**Fig. 9:**
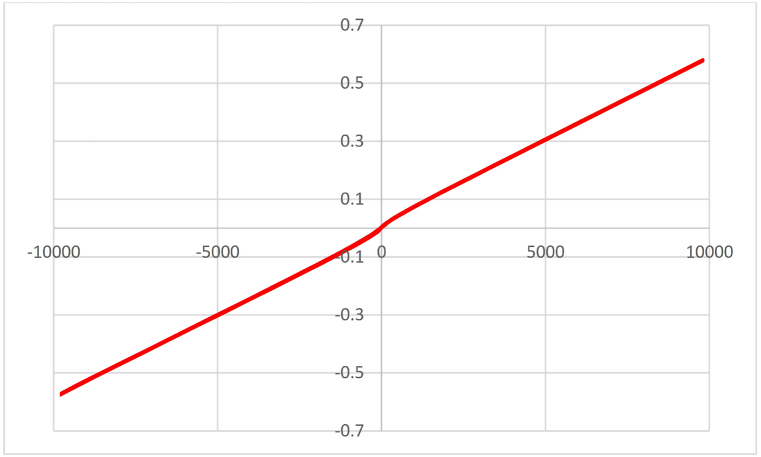
VSM curves of TiFe_2_O_4_@SiO_2_–SO_3_H.

The magnetic property of the catalyst was investigated by VSM analysis and its results are shown in Fig. 9. The value of saturation magnetism (Ms) for the catalyst is 5.51 emu/g. However, the synthesized catalyst was easily separated from the reaction mixture by an external magnet.

#### 2.3.2 Catalytic studies

##### 2.3.2.1 Esterification reactions

Next, the catalytic activity of TiFe_2_O_4_@SiO_2_–SO_3_H was investigated using the esterification of methanol with oleic acid. To optimize the reaction conditions in the presence of TiFe_2_O_4_@SiO_2_–SO_3_H for biodiesel production, the effect of various parameters such as the amount of catalyst (0.03–0.007 g), molar ratios of methanol to oleic acid, base, and temperature were investigated. By using 0.02 g of the reported nanocatalyst, the maximum production of biodiesel was obtained. A maximum conversion of 97 % (oleic acid to ester) was achieved for the temperature of 60 °C. The molar ratio between oil and methanol was considered to be 9:2 in this study for the completion of the esterification process. The excess amount of alcohol in the esterification of fatty acids will help to disperse the catalyst in the reaction media, leading to more biodiesel production. Also, excess alcohol prevents the reverse reaction and, as in other esterification reactions, more ester is produced (Table 1).

**Table 1:** Material balance calculations for the optimized yield of biodiesel in the presence of mesoporous TiFe_2_O_4_@SiO_2_–SO_3_H.

Entry^a^	Catalyst amount (g)	temperature (^◦^C)	MeOH/oleic acid molar ratio (mmol/mmol)	Time (h)	Biodiesel Produced (mg)	Unreacted Material (mg)	Biodiesel yield (%)
**1**	–	60	9:2	8	–	–	N. R
**2**	0.007	60	9:1	1.5	0.098	0.46	35
**3**	0.01	60	9:2	1.5	0.166	0.29	59
**4**	0.02	60	9:2	1.5	0.273	0.48	97
**5**	0.03	60	9:2	1.5	0.228	0.26	81
**6**	0.02	25	9:2	1.5	0.141	0.27	50
**7**	0.02	50	9:2	1.5	0.186	0.22	66
**8**	0.02	60	8:2	1.5	0.163	0.38	58
**9**	0.02	60	11:2	1.5	0.180	0.25	64
**10**	0.02	60	13:2	1.5	0.211	0.23	75

Next, the catalytic activity of TiFe_2_O_4_@SiO_2_–SO_3_H was investigated using the esterification of palmitic acid with methanol (Table 2). The model reaction was carried out in the absence of a mesoporous catalyst (TiFe_2_O_4_@SiO_2_–SO_3_H) even after 5 h, there is no product was observed. Also, according to the obtained results, the reaction efficiency decreased with the decrease in the amount of nanocatalyst. The results are shown in Table 2.

**Table 2:** Material balance calculations for the optimized yield of biodiesel in the presence of mesoporous TiFe_2_O_4_@SiO_2_–SO_3_H.

Entry^a^	Catalyst amount (g)	temperature (^◦^C)	MeOH/Palmitic acid molar ratio (mmol/mmol)	Time (h)	Biodiesel Produced (mg)	Unreacted Material (mg)	Biodiesel yield (%)
**1**	–	70	9:2	5	–	–	N. R
**2**	0.07	70	9:2	1	0.089	0.46	35
**3**	0.01	70	9:2	1	0.153	0.29	60
**4**	0.02	70	9:2	1	0.251	0.48	98
**5**	0.03	70	9:2	1	0.212	0.26	83
**6**	0.02	25	9:2	1	0.158	0.27	62
**7**	0.02	50	9:2	1	0.179	0.22	70
**8**	0.02	70	8:2	1	0.128	0.38	50
**9**	0.02	70	11:2	1	0.161	0.25	63
**10**	0.02	70	13:2	1	0.210	0.23	82

a Isolated yield.

**Scheme 4:**
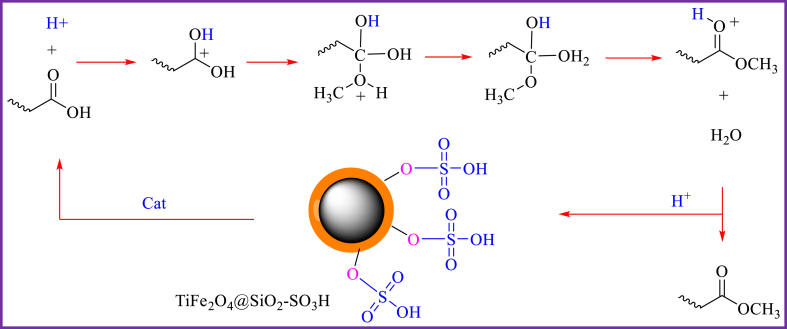
Proposed Mechanism for biodiesel in this presence of TiFe_2_O_4_@SiO_2_–SO_3_H.

The mechanism of TiFe_2_O_4_@SiO_2_–SO_3_H catalytic esterification is shown in Scheme 4. Initially, TiFe_2_O_4_@SiO_2_–SO_3_H acts as an acid catalyst to activate the carbonyl group of oleic acid to form positive carbon ions. The acidic TiFe_2_O_4_@SiO_2_–SO_3_H catalyst can provide H^+^, and the H^+^ attacks the carbonyl group of oleic acid. Then protonation of the carbonyl group leads to the generation of carbocation. In the end, after the nucleophilic attack of the methanol molecule, a tetrahedral intermediate is formed, Water molecules are removed, and finally biodiesel and H^+^ will be produced.

##### 2.3.2.2 Hot filtration

With optimal reaction conditions in hand (esterification reaction of Palmitic acid and MeOH), to test the heterogeneous nature of the TiFe_2_O_4_@SiO_2_–SO_3_H hot filtration experiment was performed under the optimal reaction conditions. In the absence of catalyst, the maximum yield was about 4% at 1h, showing that the reaction could not progress without an acid catalyst. The progress of the reaction in the presence of the acid catalyst reaches 97% after 1 h, which shows that the presence of the catalyst increases the reaction rate.

## 3 Catalyst recyclability

**Fig. 10:**
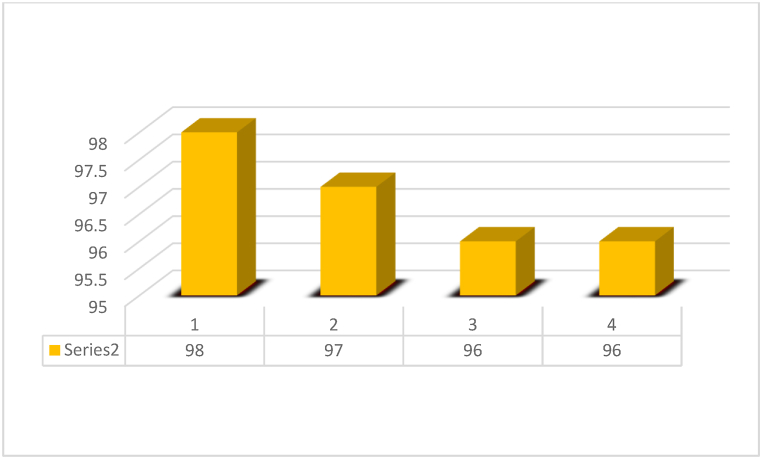
Recyclability of TiFe_2_O_4_@SiO_2_–SO_3_H.

To investigate the recyclability of the catalyst, the palmitic acid with methanol was examined as a model reaction using 0.02 g of TiFe_2_O_4_@SiO_2_–SO_3_H. Using a simple magnet, the catalyst was separated and washed several times with ethanol. The reported catalyst was recovered and reused for five periods without loss of activity (Fig. 10).

Table 3 shows the catalytic activities of previously reported catalysts compared to TiFe_2_O_4_@SiO_2_–SO_3_H in the esterification of oleic acid with methanol. Considering the reaction conditions, the reported catalysts show lower catalytic efficiency than the TiFe_2_O_4_@SiO_2_–SO_3_H catalyst. This indicates that the TiFe_2_O_4_@SiO_2_–SO_3_H catalyst is more useful in esterification reactions compared to earlier ones (Table 3).

**Table 3:** Comparison of the catalytic efficiency of reported catalysts with prepared TiFe_2_O_4_@SiO_2_–SO_3_H catalyst in the esterification of oleic acid with methanol.

Entry	Catalyst	Reaction	Time (mine)	Yield (%)	Ref
1	30% SiW11/MCM-41	Oleic acid + Methanol	1h	30	[26]
2	PCs–SO_3_H	Oleic acid + Methanol	2h	70	[27]
3	F^−^-SO_4_^2−^/MWCNTs	Oleic acid + Methanol	6h	90	[28]
4	ZrFe-SA-SO_3_H	Oleic acid + Methanol	4h	92	[29]
5	Na-Q-3T	Oleic acid + Methanol	2h	60	[30]
6	TiFe_2_O_4_@SiO_2_–SO_3_H	Oleic acid + Methanol	3h	98	This work

## 4 Conclusion

This work reports the investigation of an efficient procedure to prepare TiFe_2_O_4_@SiO_2_–SO_3_H a novel, green, magnetic catalyst. The prepared catalyst, TiFe_2_O_4_@SiO_2_–SO_3_H, was identified via BET, TEM, EDS, SEM, VSM, TGA, XRD, and FT-IR. The new catalyst was used for the synthesis of the esterification reactions. Moreover, this new TiFe_2_O_4_@SiO_2_–SO_3_H can be easily prepared from commercially available materials. Also, it can be mentioned good catalytic activity, easy separation by an external magnet, and reusability of the introduced catalyst.

## Data availability

All data generated or analyzed during this study are included in this published article [and its supplementary information files].

## CRediT authorship contribution statement

**Mohanad Yakdhan Saleh:** Data curation, Conceptualization. **Ahmed Kareem Obaid Aldulaimi:** Project administration, Methodology, Investigation, Funding acquisition. **Shakir Mahmood Saeed:** Funding acquisition, Formal analysis. **Ayat Hussein Adhab:** Writing – review & editing, Writing – original draft, Validation.

## Declaration of competing interest

The authors declare the following financial interests/personal relationships which may be considered as potential competing interests: Declaration of Competing Interest The authors declare that they have no known competing financial interests or personal relationships that could have appeared to influence the work reported in this paper. If there are other authors, they declare that they have no known competing financial interests or personal relationships that could have appeared to influence the work reported in this paper.
