# Supplementary material for: TiFe2O4@SiO2–SO3H: A novel and effective catalyst for esterification reaction
Source: Heliyon. 2024 Feb 10;10(4):e26286. doi: 10.1016/j.heliyon.2024.e26286 (PMC10875572; doi:10.1016/j.heliyon.2024.e26286)
Supplement: Multimedia component 1 [file mmc1.docx]

**TiFe_2_O_4_@SiO_2_-SO_3_H: A novel and effective catalyst for esterification reaction**

Mohanad Yakdhan Saleh^1^, Ahmed Kareem Obaid Aldulaimi^2^[[1]](#footnote-1)^*^, shakir Mahmood Saeed^3^, Ayat Hussein Adhab^4^


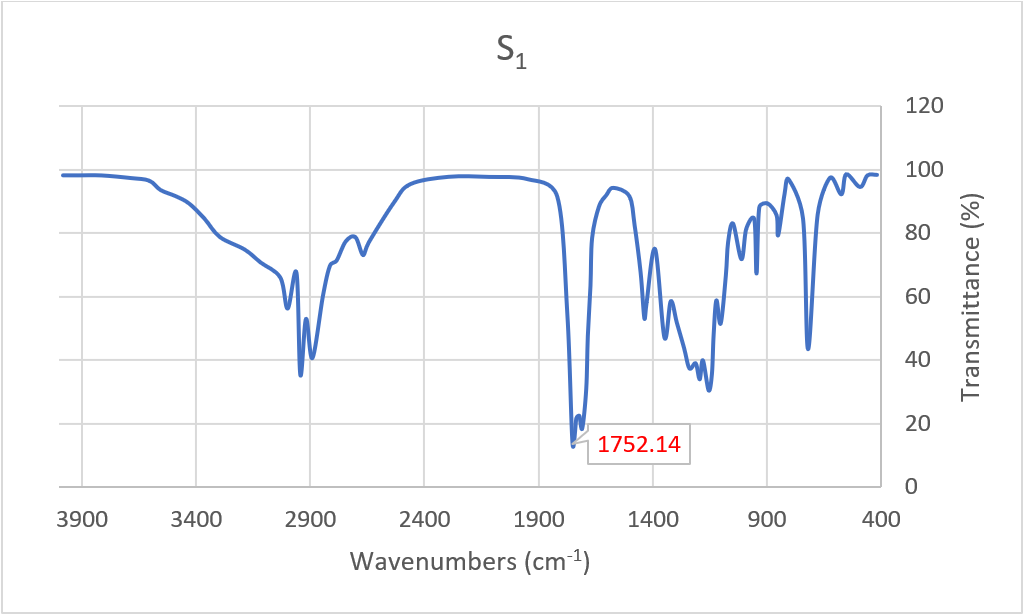


**Figure S_1_**

**Methyl oleate**: FT-IR (KBr) cm^-1^: 589, 720, 1202, 1463, 1752, 2953.


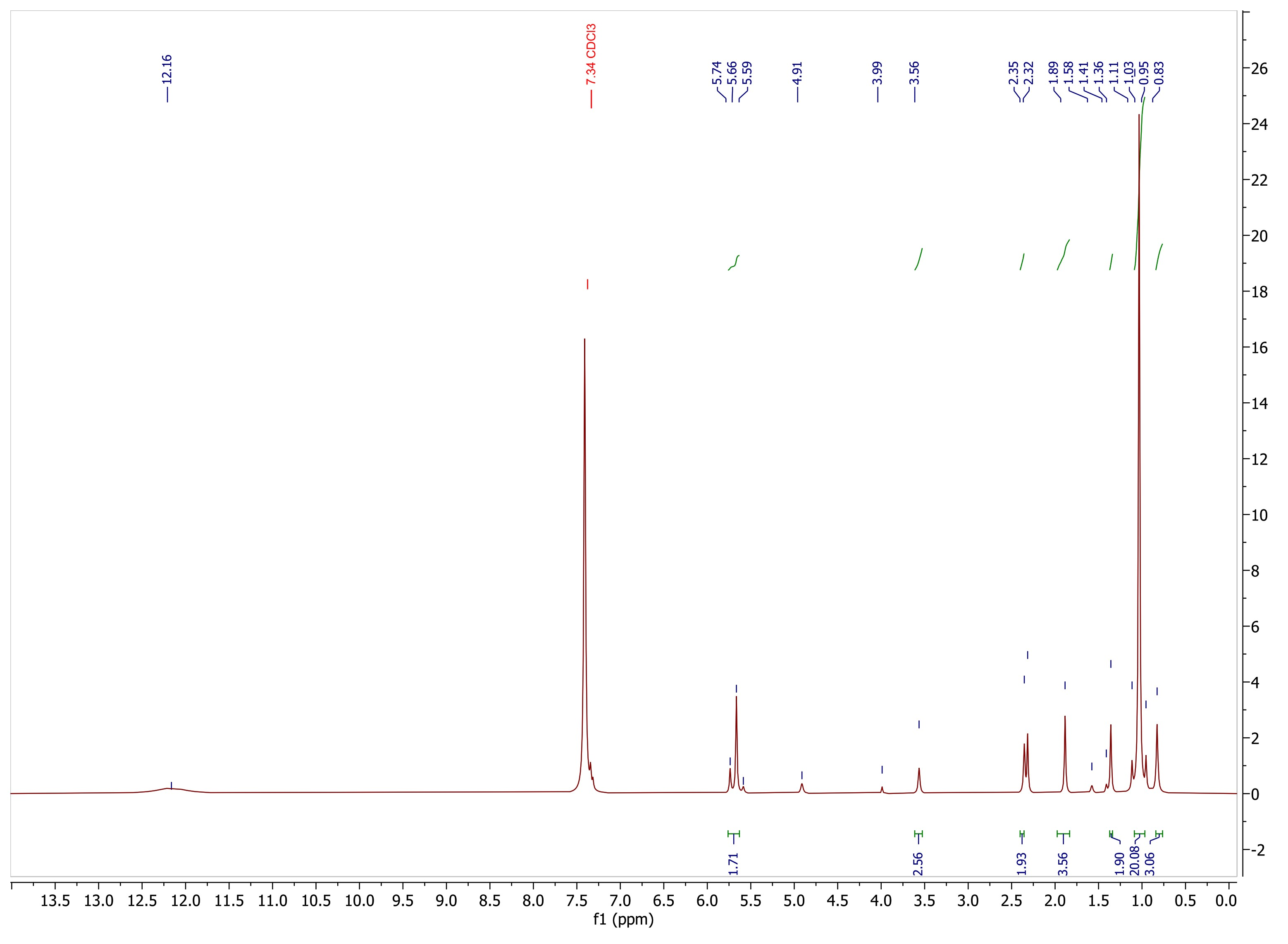


**Figure S_2_**

**Methyl oleate**:^1^H NMR (CDCl_3_, 400 MHz): δ= 0.83(s, 3H, CH_3_), 1.11(m, 20H, 10CH_2_), 1.36(m, 2H, CH_2_), 1.89 (m, 4H, 2CH_2_), 2.35(t, 2H, CH_2_), 3.56(s, 3H, CH_3_), 5.59(m, 2H, 2CH) ppm.


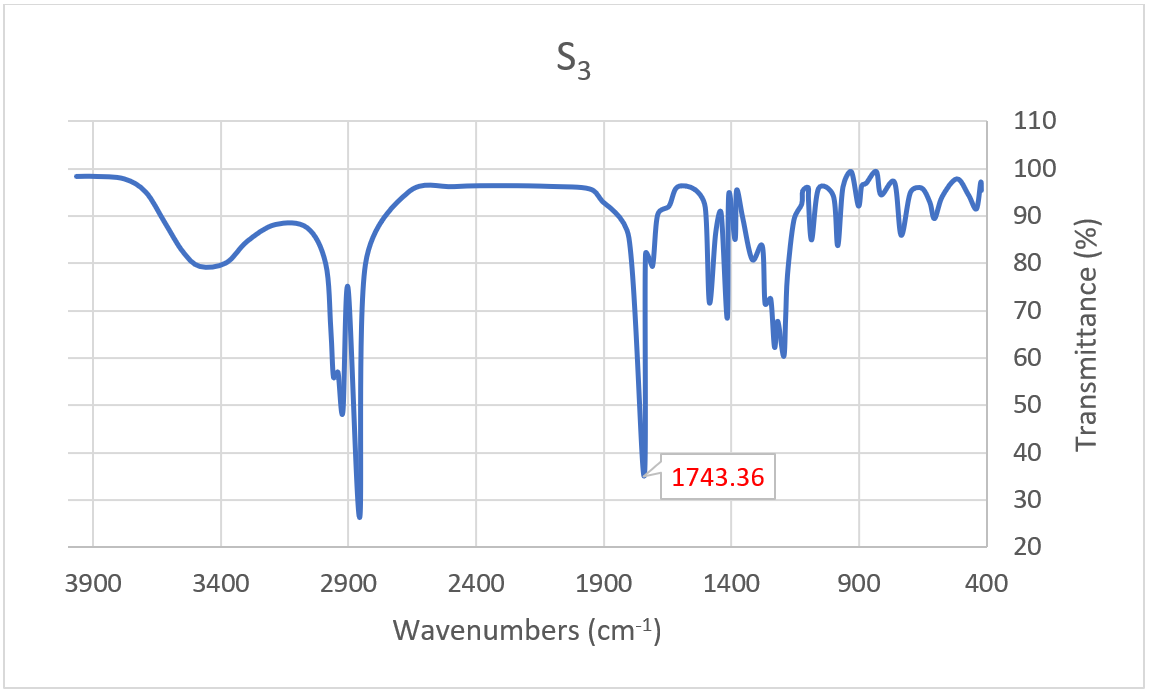


**Figure S_3_**

**Methyl stearate**: FT-IR (KBr) cm^-1^: 604, 723, 1208, 1743, 2884, 3426.


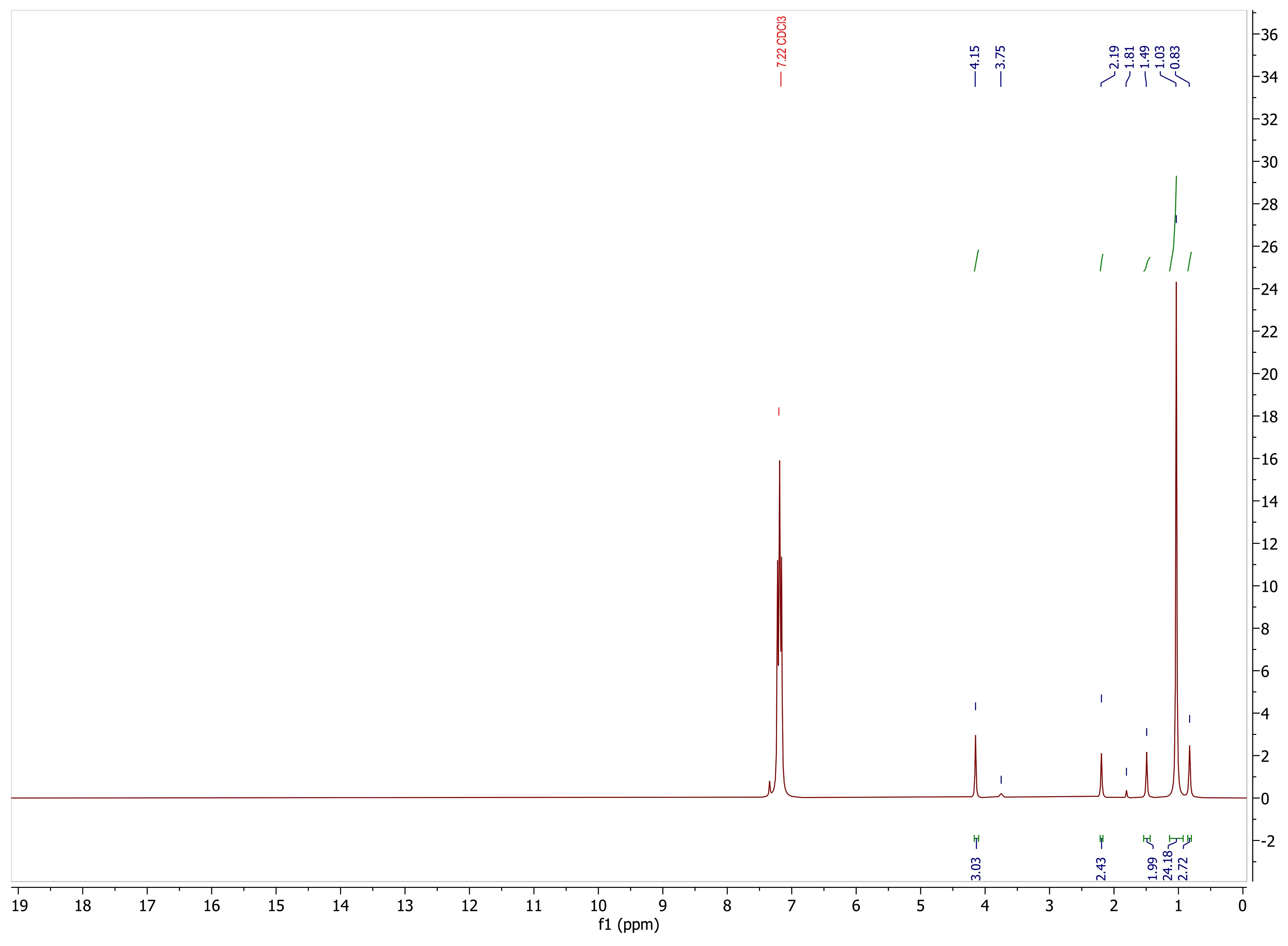


**Figure S_4_**

**Methyl palmitate**:^1^H NMR (CDCl_3_, 400 MHz): δ= 0.83(s, 3H, CH_3_), 1.03(m, 24H, 12CH_2_), 1.49(m, 2H, CH_2_), 2.19(m, 2H, CH_2_), 4.15 (s, 3H, CH_3_) ppm.

1. * Address correspondence to Ahmed Kareem Obaid Aldulaimi, College of Food Sciences, Al-Qasim Green University, Babylon, Iraq, E-mail: ahmad6565wm@gmail.com [↑](#footnote-ref-1)
